# Characterization of the Bacteriophage vB_VorS-PVo5 Infection on *Vibrio ordalii:* A Model for Phage-Bacteria Adsorption in Aquatic Environments

**DOI:** 10.3389/fmicb.2020.550979

**Published:** 2020-10-30

**Authors:** Alex Echeverría-Vega, Pablo Morales-Vicencio, Camila Saez-Saavedra, María Alejandra Alvarez, Felipe Gordillo, Rodrigo Del-Valle, Ma. Eugenia Solís, Rubén Araya

**Affiliations:** ^1^Centro de Investigación en Estudios Avanzados del Maule (CIEAM), Vicerrectoría de Investigación y Postgrado, Universidad Católica del Maule, Talca, Chile; ^2^Laboratorio de Microbiología Costera, Facultad de Ciencias del Mar y Recursos Biológicos, Universidad de Antofagasta, Antofagasta, Chile; ^3^Departamento de Matemáticas, Facultad de Ciencias Básicas, Universidad de Antofagasta, Antofagasta, Chile; ^4^Centro de Biotecnología de los Recursos Naturales, Facultad de Agronomía y Ciencias Forestales, Universidad Católica del Maule, Talca, Chile; ^5^Departamento de Matemática, Física y Estadística, Facultad de Ciencias Básicas, Universidad Católica del Maule, Talca, Chile; ^6^Instituto de Ciencias Naturales Alexander von Humboldt, Facultad de Ciencias del Mar y Recursos Biológicos, Universidad de Antofagasta, Antofagasta, Chile

**Keywords:** bacteriophage adsorption dynamics, model for bacteriophage infection, phage-host interaction, particle collision, phage therapy

## Abstract

A mathematical first-order difference equation was designed to predict the dynamics of the phage-bacterium adsorption process in aquatic environments, under laboratory conditions. Our model requires knowledge of bacteria and bacteriophage concentrations and the measurements of bacterial size and velocity to predict both the number of bacteriophages adsorbed onto their bacterial host and the number of infected bacteria in a given specific time. It does not require data from previously performed adhesion experiments. The predictions generated by our model were validated in laboratory. Our model was initially conceived as an estimator for the effectiveness of the inoculation of phages as antibacterial therapy for aquaculture, is also suitable for a wide range of potential applications.

## Introduction

Viruses infecting bacteria, known as bacteriophages or phages, are the most common and diverse entities in the biosphere, especially in aquatic environments (Breitbart and Rohwer, 2005; Suttle, 2005). In the ocean, its numbers are generally near 10 millions of particles per mL of water, a value that decreases with water depth, coastal distance depth, and coastal distance (Cochlan et al., 1993; Paul et al., 1993). Its major abundance has been observed in coastal sediments where their numbers can reach up to one billion per mL (Bergh et al., 1989; Paul et al., 1993; Hewson et al., 2001). Besides this, they are important for mobilizing and transferring genetic information (Abedon, 2008), and promoting the evolution of organisms. Phage-host interactions are models of synergic evolution with a vast population size and a short duplication time. This fact makes these systems ideal for the study of evolution and community-dynamics and for the quick observation of the ecological principles (Lenski and Levin, 1985; Weitz et al., 2005; Brockhurst et al., 2007).

Félix d’Herelle discovered bacteriophages in 1917 and immediately visualized their potential to control bacterial diseases (D’Herelle, 1917). Nowadays, due to the excessive use of antibiotics (Blazquez et al., 2002; Harbottle et al., 2006; Madhusudana Rao and Lalitha, 2015), the strategy of using lytic bacteriophages as antimicrobial agents, has become an interesting alternative for controlling bacterial infections (Matsuzaki et al., 2005; Almeida et al., 2009; Carvalho et al., 2012; Verbeken et al., 2014; Keen and Adhya, 2015; Kutter et al., 2015; Sarhan and Azzazy, 2015). The main advantages of bacteriophage usage are their high specificity (even at strain level), infective ability, and their exponential growth curve in the presence of a target bacteria, with a higher efficiency than antibiotics (Levin and Bull, 1996). According to Drake et al. the mutation rates of bacteriophages are of three orders of magnitude higher than bacteria (Drake et al., 1998), which means, they have the potential to overcome the bacterial resistance mechanism (Buckling and Rainey, 2002; Brockhurst et al., 2007). However, there are diverse strategies of bacteriophage infection and bacterial resistance, allowing for their natural coexistence and equilibrium both at ecological (Schrag and Mittler, 1996; Rabinovitch et al., 2003; Heilmann et al., 2012) and genomic levels (Buckling and Rainey, 2002; Barrangou et al., 2007; Stern and Sorek, 2011; Samson et al., 2013). However, if we want to apply methodologies based on bacteriophages as biological controllers, it is important to understand the dynamics that guide the phage-host interaction.

The law of mass action, particle collision, and diffusion of the virion are the determinant factors for the collision and adsorption of bacteriophages onto bacterial surfaces (Koch, 1960; Stent, 1963; Payne and Jansen, 2000; Buckling and Rainey, 2002; Moldovan et al., 2007; Cairns et al., 2009) are used in most mathematical approaches. The more accepted models define the initial adsorption as an irreversible process, which depend on the time (Gani, 1965; Chang and Chang, 1969; Murray and Jackson, 1992; Carletti, 2007; Stopar, 2008; Cairns et al., 2009) and are based on the Poisson distribution (Stent, 1963; Stopar, 2008). The determining factors for these models are bacteriophage and bacterial density (Beretta and Kuang, 1998; Payne and Jansen, 2001; Cairns et al., 2009), temperature (Moldovan et al., 2007), and the presence of cellular receptors (Schwartz, 1976; Moldovan et al., 2007) among others. However, some of these models minimize the surrounding space so that the bacterium is unable to reach and influence due to its swimming movements and the size of the area (Yassky, 1962; Gani, 1965; Lowe et al., 1987; Moldovan et al., 2007). Finally all of these deviations were commonly fixed through the generation of a phage adsorption rate constant (*k*) *a posteriori*, performed in a set of *in-vitro* experiments (Hyman and Abedon, 2009). On the other hand, the probability of an encounter between a bacteriophage and its host has been attributed to factors such as abundance, movement, shape, and size (Murray and Jackson, 1992; Payne and Jansen, 2001). The size and number of the host defines the quantity of the occupied space in a determined volume, which logically establishes the probability of a bacteriophage-host encounter. Besides, if one considers, that some bacteria possess high motility in comparison to the diffusion coefficients of virion particles, it is imperative to include this factor in the calculation of the initial adsorption. It is estimated that in seawater, it is possible to find a mean value of 1 × 10^6^ bacteria per mL (Cochlan et al., 1993). This means that the available space for each one of them is around 0.1 μL (1 × 10^–6^ mL).

Previous research has show that bacteriophages are present in an average concentration of one magnitude order over the total amount of bacteria (Wommack and Colwell, 2000), and up to three magnitudes of order as the maximum in highly populated areas (Bergh et al., 1989; Paul et al., 1993; Hewson et al., 2001). This means that a planktonic bacterium could theoretically find one phage virion every 10 μm in its traveling path when moving in any direction. Some marine bacteria can exceed the speed of 300 μm/s (Mitchell et al., 1995), in that case it would find a total of 100,000 bacteriophages per hour. On the other hand, the movement of a bacteriophage only depends on its diffusion coefficient that, in general, has a value close to 5 × 10^–8^ cm^–2^s^–1^ (Murray and Jackson, 1992), which, in most cases, is negligible compared to the speed of motile bacteria (Joiner et al., 2019).

In this work, we have designed and evaluated a model based on the interaction dynamics involving the strictly lytic bacteriophage vB_VorS-PVo5 (PvB) and its host bacteria *Vibrio ordalii* ATCC-33509 (VO). VO is an important pathogen that causes great losses for the aquaculture industry (Schiewe et al., 1981). Their specific phage (PvB) is important because of its potential to be used as an antibacterial agent in phage therapy in aquaculture (Echeverría-Vega et al., 2016). It was isolated from mollusk inhabitants of the Antofagasta coast (Chile). Our model was developed after analyzing the variation and recalculation of the probability of encounters between phages and bacteria in discrete time periods. It is possible to model the adsorption process by using the measurements of the initial parameters such as the total amount of initial bacteriophages, velocity, size, and number of bacteria and thus, we can predict both the number of adsorbed phages and infected bacteria at the same time. Unlike the classical adsorption theory (Hyman and Abedon, 2009) which determinates an adsorption constant *a posteriori* through a battery of previous tests, our model uses only the measured physical parameters of the particles involved (phages and bacteria) to model the adsorption *a priori*. As a result, it also reduces the risk of experimental errors. The proposed model fits with the data measured and its construction is based on the probability of a collision between particles, therefore it could be applied to other phage-bacterium systems and can be extrapolated to predict any other phenomena involving particle collisions.

## Materials and Methods

### Bacterial Strain and Growth Conditions

A Chilean wild-type of the flagellated Gram-negative *Vibrio ordalii* (VO) was used for this study. VO is a marine pathogenic bacterium originally isolated from infected *Argopecten purpuratus* (Lamarck 1819) larvae (Riquelme et al., 1995) and have a high impact on the salmon farming industry. VO was routinely grown and maintained in a liquid tryptic soy broth (TSB) and trypticase soy agar (TSA) supplemented with a 50 % total volume of sterile seawater at 25 °C (Oxoid, United States).

### Culture of Phage and Bacteria

The phage vB_VorS-PVo5 (PvB) is described as a lytic strain belonging to the Siphoviridae family (Echeverría-Vega et al., 2016). It was obtained from soft tissues of *Perumytilus purpuratus* (Lamarck 1819) recovered from the coast of Antofagasta, Chile using a previously described methodology (Echeverría-Vega et al., 2019).

### Transmission Electron Microscope Analysis (TEM)

All the preparations was performed following the protocol described by Carlson (Carlson, 2005) with some slights modifications. Stabilized carbon formvar grids (EMS, United States) were used to mount the phage samples for TEM. A drop (10 μL) of the sample containing a viral concentration greater than 1 × 10^8^ viral particles per milliliter (VP/mL) was placed on the grid. After 15 min the liquid was removed with an absorbent paper. Subsequently, 10 μL of a staining reagent solution (1 % Uranyl Acetate in water) was placed on the grid for 15 min. Excess water was removed with blotting paper and then, allowed to air dry for 15 min. The samples were observed using a Phillips 100 CM TEM.

### Setting of Initial Parameters

Counting the bacterial number and size was completed by the DAPI-staining method (Porter and Feig, 1980). For this, 400 cells for a sample were counted in triplicate. The bacterial size was obtained from the mean of several measurements of 100 cells, using images obtained from the Zeiss Axiolab fluorescence microscope (Zeiss, Germany). The bacterial volume (*V*_*B*_) was calculated considering the cells as cylindrical by using the formula V = πr^2^l, where r is the radius and l is the length. The cell including the flagellum has a length of 13 μm (Schiewe et al., 1981) and *Vibrio* oscillates with a wavelength of 3,2 μm and can turn in angles up to 90° when tumbling (Xie et al., 2011). Bacteria can tumble every 1 s in average and furthermore generate turbulences in the medium when rotating (Joiner et al., 2019). Then, bacteria are likely to interact with phages over a space beyond their place of positioning. All these factors were taken into account while defining the “Vital space” (*V*_*S*_) which is the entire potential coverage zone where the bacterium can interact with the phage or the volume that the bacterium is capable of reaching when it is in a determined space in a specific period of time. Our hypothesis is that *V*_*S*_ is one spherical volume with a radius of 5 times the size of Δ*l*_*B*_ surrounding each bacterium. Bacteriophage number counting was achieved using the method of double-layer agar (Carlson, 2005; Kropinski et al., 2009). For that purpose, a triplicate of four dilutions were performed in TSB medium (1/10, 1/100, 1/1000, and 1/10000) containing the bacteriophage. The plates were incubated overnight to perform the counting of plaques by visual inspection the next day. The measurements of the bacteriophage size were completed by transmission electron microscopy (TEM) images. The bacteriophage volume (P_*B*_) was calculated considering the head as a sphere and the tail as a cylinder. For the measurements of bacterial movement, one sample was taken from the liquid culture in the exponential phase (16 h after the renovation) and then it was observed under the Zeiss Axiolab microscope (Zeiss, Germany) equipped with an objective grid with 100 squares of 100 μm^2^ each. One hundred measurements of the time taken by one bacterium to cross a zone of 20 μm was carried out to calculate bacterial velocity. For the bacteriophage movement, a diffusion constant of 5 × 10^–8^ cm^–2^s^–1^ was considered (Murray and Jackson, 1992).

### Adsorption Curves and Determination of Growth Parameters

A bacterial growth curve was generated with the data obtained after measuring the turbidity of the liquid cultures by UV-visible spectrophotometry in triplicate. To determine the effect of the phage on bacterial growth, VO was inoculated in 500 mL of TSB medium with a final concentration of 5 × 10^6^ colony-forming units per milliliter (CFU/mL) and distributed in 72 glass tubes. An inoculum of 5 × 10^7^ plaque-forming units per milliliter (PFU/mL) of bacteriophage was added to 36 tubes to obtain a multiplicity of infection (MOI) = 10. During the first hour of cultivation, the absorbance was measured every 15 min. Then, from the second hour to the fifth and a half hour, it was measured every 30 min. A final measurement was taken after 24 h. To determine the latency period and the burst-size of the phage under study, the growth curve was achieved using the one-step methodology (Carlson, 2005; Hyman and Abedon, 2009). For this, 1 × 10^7^ CFU/mL of VO with a total of 1 × 10^2^ PFU/mL of PvB was used as the inoculum. A 100 μL sample was collected after every 15 min to grow and later count the phage plaques in double agar plates. The latency time is defined as the period in which there is no significant variations in the number of phages (slope = 0). The burst-size was calculated by dividing the number of total phages obtained in the beginning of the logarithmic phase with the initially added phages. In order to empirically determine the rate of the adsorption of a phage, the previously described established protocols (Hyman and Abedon, 2009; Kropinski, 2009) were performed with some modifications. A VO liquid culture was grown at 25°C in TSB medium supplemented with 50 % sterile marine water, an inoculum of 1 × 10^8^ CFU/mL was added after 24 h in the exponential phase. Triplicate assays using two different dilutions (1 × 10^6^ and 1 × 10^5^ CFU/mL) of this bacterial culture in the exponential phase were performed in 50 ml plastic tubes. Two different concentrations of bacteriophages (8000 and 800 PFU/mL) were added. Then, we took 1 mL samples once every minute for a total time of 10 min and transferred them to a sterile tube containing chloroform to lyse the bacteria. Samples were agitated for 10 s in a vortex, and the supernatant was immediately extracted to count the free phage virions according to the methodology described above.

### Modeling of the Adsorption Process

The simulation and graphics were completed using the Maple software (Maplesoft, Cybernet Systems Co., Ltd., Japan) and Microsoft Excel software (Microsoft Office, United States). The simulations consider:

– Infected bacteria, increasing the number of bacteria and phages keeping the MOI = 1.
– Infected bacteria over time, maintaining the bacterial number at 10^6^ CFU and the MOI > 1.
– Infected bacteria by varying its speed, maintaining the bacterial concentration in 1 × 10^6^ CFU and MOI = 1.
– Adsorbed phages, maintaining constant the number of phages at 1 × 10^6^ UFP/mL and by varying the bacterial number.

We performed adsorption experiments using the previously described protocol and default parameters with both 8000 and 800 phages, respectively. We plotted the adsorption curves comparing with both the classical adsorption model (Hyman and Abedon, 2009) and a cubic polynomial regression model which best fit the experimental data. We evaluated the predictability of our model comparing the experimental curves with those generated by simulations. This model was deposited into BioModels (Chelliah et al., 2014) and assigned the identifier MODEL2007190001.

## Results

**FIGURE 1:**
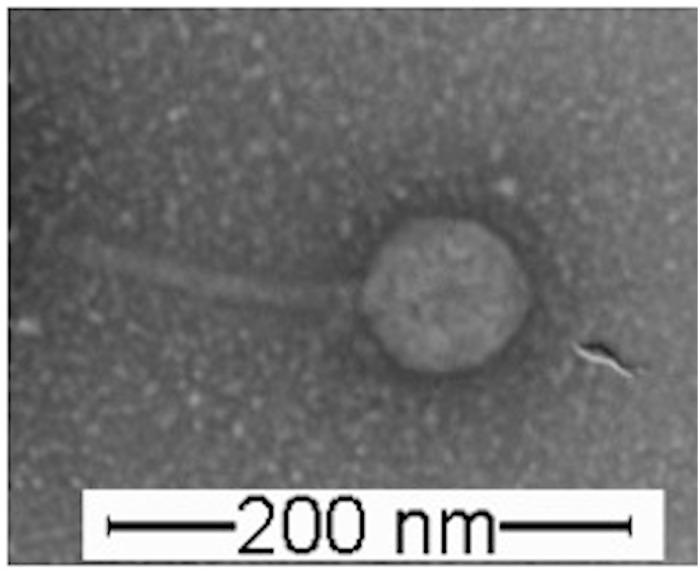
Observation of the viral particles in electronic transmission microscopy (TEM).

The phage PvB has been described in previous studies (Echeverría-Vega et al., 2016) as a strain belonging to the Siphoviridae family (Order Caudovirales), which groups viruses containing double-stranded DNA chains. It produced lysis plaques of ∼ 2 mm of diameter in the agar plate when infected with VO. The observation of the viral particles in electronic transmission microscopy revealed an icosahedral capsid of ∼ 85 nm of diameter and a tale of ∼ 150 nm long, which gives a volume of 2,1 × 10^–17^ mL (Figure 1).

**FIGURE 2:**
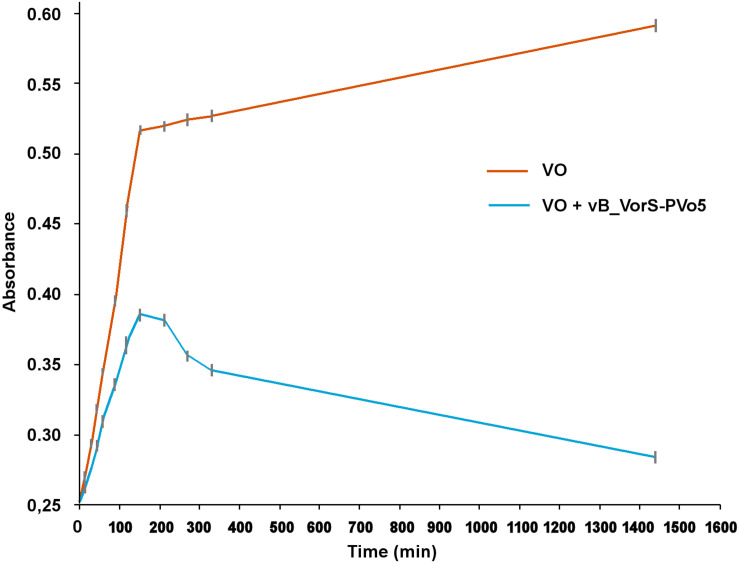
Growth curve of VO in absence and presence of PvB.

The bacterial length for VO is approximately 1.8 μm with 0.6 μm of width and its calculated volume is 5 × 10-13 mL (*V*_*B*_) which is equivalent to a sphere of 1 uL diameter (Δ*l*_*B*_). Vital space *V*_*S*_ was calculated as 5 × 10-10 mL. The bacterial mean velocity was 30 μm/s (SE = 0.49). The growth test showed a strong diminishing of VO growth when PvB was inoculated to the culture, which indicated a clear inhibitory effect of the phage on the bacterial population after 120 min, VO lysis still increased until 1,450 min (Figure 2).

**FIGURE 3:**
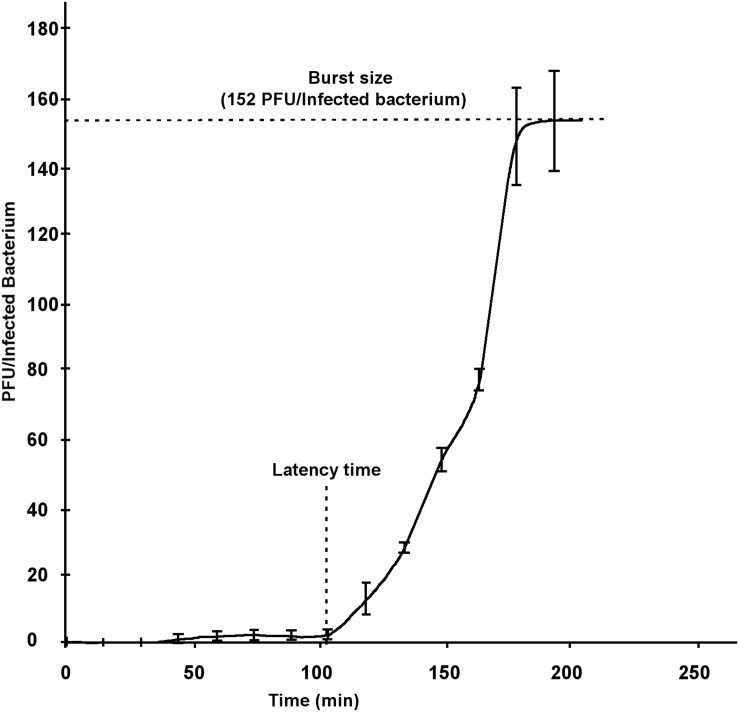
One-step growth curve of PvB.

The corresponding measurements were taken on the growth curve of PvB by the one-step method. The lapsed time between the adsorption and the bacterial lysis (latency time) was estimated as 100 min and the burst-size was around 152 phages/infected-bacteria. These parameters were considered in the typical range for a Siphoviridae family member (Yu et al., 2013) and allowed us to use it for further tests oriented to the bacterial control (Figure 3).

Our proposed model of adsorption considered the established theory according to which the encounters should randomly occur as per the Poisson equations (Ellis and Delbrück, 1939). If adsorption is considered as a dependent function of the space, in which phage and bacteria are found, the total volume of the liquid medium in which they cohabit, will be understood as an integration of spaces of the size of one bacterial vital space (*V*_*S*_). In this way, the probability of the encounter of a phage with the bacteria is computed according to the total number of spaces.

We define “Total Slot” (*E*_*T*_) as the theoretical number of bacterial vital spaces that fits in the total volume, in other words, the total volume used in the cell culture divided by the volume of one vital space (Equation 1).

(1) $$E_{T}=\frac{V_{T}}{V_{S}}$$

*E*_*T*_: total slots.*V*_*T*_: total volume.*V*_*S*_: bacterial vital space.

For the case of VO, the *V*_*S*_ is 5 × 10^–10^ mL, which means in 1 mL, there is around 2 × 10^9^ *E*_*T*_ available.

If an amount of phage (insignificant volume of 2x10^–17^ mL) is added (*F*_*T*_) to a known volume of a liquid medium (*V*_*T*_), it will be distributed using probability as defined according to the Poisson equation. Thus, the event *X* to find *n* phages in one slot *E* is defined by the probability:

(2) $$P\left(X=n\right)=\frac{e^{-\lambda }\cdot \lambda^{n}}{n!}$$

where λ is the average of phages in a slot (*E*).

If *n=0* and λ = *F*_*T*_(1/*E*_*T*_), then *P*_0_=*P*(*X* = 0) is the probability of not finding phages in one slot and it is defined by:

(3) $$P_{0}=e^{-F_{T}/E_{T}}$$

Then, the probability of finding at least one phage in one slot (or simply adsorption probability) is:

(4) $$P_{a}=1-e^{-F_{T}/E_{T}}$$

*P*_*0*_: probability that no phage is in one place.

*P*_*a*_: probability that at least one phage fits in a punctual slot = (1−*P*_0_).

*F*_*T*_: total phage added initially.

*E*_*T*_: total slots.

Bacterial velocity is a critical parameter in this process. If we know the bacterial velocity (υ_*B*_), we can define the interval of time used by one bacterium to move a segment of length Δ*l*_*B*_, equivalent to the diameter of one sphere of the same volume of the bacterium and it is defined as:

(5) $$\Delta \,t=\frac{\Delta \,l_{B}}{\upsilon_{B}}$$

The averages for VO are: Δ*l*_*B*_ = 1 μm and υ_*B*_ = 30 μm/s. Therefore Δ*t* = 1/30 s and the time taken by VO to move from *E* to *E*′ (or *E*_(*t*)_) to *E*_*t* +Δ*t*_) is 0.033s approximately.

Considering initial conditions in *t*_*0*_, we can define the steps of our discrete model as follows:

(6) $$t_{k}=t_{0}+k\,\Delta \,t,$$

where *k* = 1, 2, 3,…, and therefore we define the variables as:

*E*_*t_k_*_: number of slots with phages at time *t*_*k*_.

*F*_*t_k_*_: number of free phages at time *t*_*k*_.

*B*_*t_k_*_: number of bacteria with adsorbed phages measured at time *t*_*k*_.

Using these variables, we can write the model as:

(7) $$E_{t_{0}}=E_{T}\cdot P_{a},$$

(8) $$F_{t_{0}}=F_{T}\,\left(1-\frac{B_{T}}{E_{T}}\right),$$

(9) $$B_{t_{0}}=B_{T}\cdot P_{a}$$

Equations 7, 8, and 9 show that at the initial time t_0_, adsorption probability determines the number of slots occupied with phages. Its initial number and the proportion of empty slots (without bacteria) determine the number of virions (free phages). On the other hand, the number of bacteria multiplied by the probability of adsorption generates the number of infected bacteria (with at least one phage adsorbed).

**FIGURE 4:**
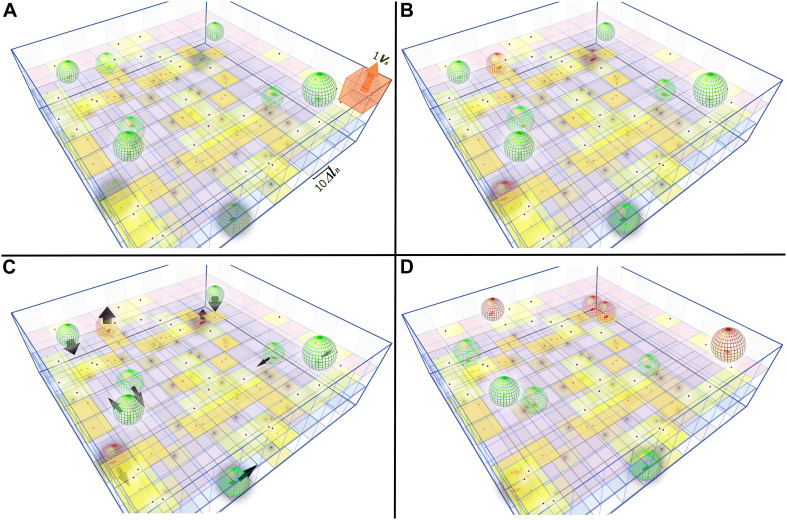
Schematic 3D representation of the phage-bacteria adsorption process. A grid of 100x100x3 squares represent the total space (*E*_*T*_). The sides of squares are 10Δ*l*_*B*_. One vital space (*V*_*S*_) is the spherical volume that fits inside one cube (represented in **A**). Red dots on yellow squares represent slots with phages. **(A)** Total phages (F_*T*_ = 100) are distributed randomly across the space (sizes are not in scale), green ellipses are uninfected bacteria with their vital space represented by a green mesh sphere*V*_*S*_. **(B)** When one bacterium arrives in a yellow slot (with at least one phage inside), it is infected and turns into red. The phages in these vital spaces are adsorbed. **(C)** Bacteria move 10Δ*l*_*B*_ in random directions, which is indicated by black arrows. **(D)** Previously infected bacteria (red colored) are able to gain more adsorbed phages when moving to a new infected slot. All the uninfected bacteria that move to an infected slot become infected. A dark halo surrounding bacteria and phages, indicates they are located on the lower level and a white halo indicates they are on the upper level.

To explain the model in a very simplistic way, in Figure 4 we can see a total volume *V*_*T*_ divided in cubes representing the total space (*E*_*T*_ = 300). If we add *F*_*T*_ = 100 phages, the probability that one slot contains phages (*P*_*a*_) is 28% and its distribution is shown in Figure 4A. The probability of a phage not fitting in an empty slot (*P*_*0*_) is 72% (Eq. 4). If *B*_*T*_ = 10, the probability that a bacteria fits in the infected place is given by *P*. We get the initial number of infected bacteria *B*_*t*_0__ = 3 by multiplying *P*_*a*_ with the bacterial number (Eq. 9). We also get free phages in *t*_*0*_ (*F*_*t*_0__ = 67) from Eq. 8 (Fig. 4B). For each time interval “Δ*t*”, the bacteria will move 1Δ*l*_*B*_. Figure 4C shows 10 time intervals. This new positioning of bacteria generates a new adsorption probability (Figure 4D), that is:

(10) $$B_{t_{1}}=B_{t_{0}}+\left(B_{T}-B_{t_{0}}\right)\,\frac{E_{t_{1}}}{E_{T}},$$

and

(11) $$F_{t_{1}}=F_{t_{0}}\,\left(1-\frac{B_{T}}{E_{T}}\right)$$

Once one bacterium moves from one slot to another, it carries the adsorbed phages along with it and thus leaves the slot empty where the phages were previously. This diminishes the probability that one bacterium is infected the next time (*t*_*k+1*_). Besides, it is feasible that this bacterium would continue adsorbing phages when it moves. Then, the number of infected bacteria in each interval depends on the number of slots containing phages in each time-lapse (Δ*t*). From this, the followings points can be inferred:

– The total infected bacteria in a time lapse Δ*t* will be the sum of the bacteria that was placed in an infected slot in time *t*_*k–1*_, and
– The total amount of infected bacteria in *t*_*k*_ will be the total of infected bacteria in time t_*k–1*_ plus the ones that were infected between time *t*_*k–1*_ and *t*_*k*_. This new number of infected bacteria is calculated by multiplying the number of not infected (free) bacteria with the ratio between total slots with phages in *t*_*k*_ (*E*_*t_k_*_) and total slots (*E*_*T*_).
– The total amount of free bacteria is calculated as the difference between total bacteria and those infected in *t*_*k–1*_.

This can be summarized in the following equation:

(12) $$B\,t_{k}=B_{t_{k-1}}+\left(B_{T}-B_{t_{k-1}}\right)\,\frac{E_{t_{k}}}{E_{T}}$$

This shows that there is a clear dependency on infected bacteria within the space. The total slots with phages which remain for the next time-interval (*E*_*t_k_*_) are the slots with phages in *t*_*k*−1_, minus the probability that one bacterium moves to a slot with phages (Eq. 7). No limit in the number of adsorbed phages on each bacteria was considered. Therefore we have the following equations:

(13) $$E_{t_{k}}=E_{t_{k-1}}\,\left(1-\frac{B_{T}}{E_{T}}\right)$$

And for phages:

(14) $$F_{t_{k}}=F_{t_{k-1}}\,\left(1-\frac{B_{T}}{E_{T}}\right)$$

where $\frac{B_{T}}{E_{T}}$ correspond to the *per-capita* availability of slots *E* and free phages *F* in a period of time Δ*t* (i.e., $\frac{\Delta \,N}{N\,\Delta \,t}$for *N=E* or *N=F*). From equations 7, 8, 9, 12, 13, and 14 we can see the following equations system:

(Eq. System 1) $$\begin{array}{cc} B_{t_{k}}=B_{t_{k-1}}+\left(B_{T}-B_{t_{k-1}}\right)\,\frac{E_{t_{k}}}{E_{T}},B_{t_{0}}=B_{T}\cdot P_{a} & \\ E_{t_{k}}=E_{t_{k-1}}\,\left(1-\frac{B_{T}}{E_{T}}\right),\;E_{t_{0}}=E_{T}\cdot P_{a} & \\ F_{t_{k}}=F_{t_{k-1}}\,\left(1-\frac{B_{T}}{E_{T}}\right),\;F_{t_{0}}=F_{T}\,\left(1-\frac{B_{T}}{E_{T}}\right) & \end{array}$$

Furthermore, from equation 13 and 14 we calculated their explicit solutions:

(15) $$E_{t_{k}}=E_{T}\,P_{a}\,{\left(1-\frac{B_{T}}{E_{T}}\right)}^{k}$$

and

(16) $$F_{t_{k}}=F_{T}\,{\left(1-\frac{B_{T}}{E_{T}}\right)}^{k+1}$$

By combining, we can define slots in term of phages:

(17) $$E_{t_{k}}=E_{T}\,P_{a}\,\frac{F_{t_{k-1}}}{F_{T}}$$

With that, Eq. System 1 can be reduced to a first order system with two states (*B* and *F*) given by:

(Eq. System 2) $$\begin{array}{cc} B_{t_{k}}=B_{t_{k-1}}+\left(B_{T}-B_{t_{k-1}}\right)\,P_{a}\,\frac{F_{t_{k-1}}}{F_{T}},B_{t_{0}}=B_{T}\cdot P_{a} & \\ F_{t_{k}}=F_{t_{k-1}}\,\left(1-\frac{B_{T}}{E_{T}}\right),\;F_{t_{0}}=F_{T}\,\left(1-\frac{B_{T}}{E_{T}}\right) & \end{array}$$

### Phage-Bacteria System Analysis

#### Adsorption Time

From Eq. 6, the system times are given by *t*_*k*_ = *t*_0_ + *k*Δ*t*, so it is possible to determine the time when there were no more free phages (or when they were all absorbed by bacteria), that is, we can calculate the value of *K* as the number of approximate time periods at which the system stabilizes. From the Eq. 16, we look for *F*_*t_k_*_ < 1, and thus we obtain the inequality:

(18) $$K>-\frac{I\,n\,\left(F_{T}\right)}{I\,n\,\left(1-\frac{B_{T}}{E_{T}}\right)}-1$$

and if:

(19) $$h=-\frac{I\,n\,\left(F_{T}\right)}{I\,n\,\left(1-\frac{B_{T}}{E_{T}}\right)}-1$$

then, *K* is the lowest integer greater than *h*. As *K* gives the number of time intervals that must occur until 0.99 phages remains, the number of phages will be approximated to the nearest greater integer, i.e., > 0.5, considered as one unit. Then the new value of *K* is obtained from *F*_*t_k_*_ < 1/2, resulting in:

(20) $$K>-\frac{I\,n\,\left(2\,F_{T}\right)}{I\,n\,\left(1-\frac{B_{T}}{E_{T}}\right)}-1$$

#### Incorporating MOI

If the multiplicity of infection (*MOI* = *F*_*T*_/*B*_*T*_) is incorporated into the model, we get a reinterpretation of Eq. System 2, as:

(Eq. System 3) $$\begin{array}{cc} B_{t_{k}}=B_{t_{k-1}}+\left(B_{T}-B_{t_{k-1}}\right)\,P_{a}\,\frac{F_{t_{k-1}}}{F_{T}},B_{t_{0}}=B_{T}\cdot P_{a} & \\ F_{t_{k}}=F_{t_{k-1}}\,\left(1-\frac{F_{T}/E_{T}}{M\,O\,I}\right),\;F_{t_{0}}=F_{T}\,\left(1-\frac{F_{T}/E_{T}}{M\,O\,I}\right) & \end{array}$$

#### Stationary Solutions

As Eq. system 3 is a two-dimensional discrete model, the study of its dynamics is subject to determining its equilibrium solutions or equilibrium points and its associated stability. We understand that for the equilibrium solution of any pair *B*^∗^, *F*^∗^ that *B*_*t_k_*_ = *B*^∗^ and *F*_*t_k_*_ = *F*^∗^, for all *K* ≥ 0 so that stationary and constant system solutions are available. Then from the Eq. System 3, we propose the following option:

(21) $$B^{*}=B^{*}+\left(B_{T}-B^{*}\right)\,P_{a}\,\frac{F^{*}}{F_{T}}$$

and

(22) $$F^{*}=F^{*}\,\left(1-\frac{F_{T}/E_{T}}{M\,O\,I}\right)$$

after solving it we get

(23) $$\left(B_{T}-B^{*}\right)\,F^{*}=0$$

and

(24) $$F^{*}\,\left(\frac{F_{T}/E_{T}}{M\,O\,I}\right)=0$$

From the resolution of Eq 23 and Eq 24, the system has infinite stationary or equilibrium solutions of the form *e* = (*B*^∗^, 0), with *B*^∗^ ≤ *B*_*T*_, that is, solutions showing that all or part of the total bacteria could be infected after the k^*th*^ time step in which the phages are depleted. For the stability analysis of the equilibria, we will use the classic theory of determination of the eigenvalues of the Jacobian matrix *J*, where *J* is obtained by the partial derivatives with respect to phages and bacteria on the right side of Eq. system 3, that is:

(25) $$J\,\left(B_{t_{k-1}},F_{t_{k-1}}\right)=\left(\begin{array}{cc} 1-\frac{P_{a}\,F_{t_{k-1}}}{F_{T}} & \left(B_{T}-B_{t_{k-1}}\right)\,\frac{P_{a}}{F_{T}}\\ 0 & 1-\frac{F_{T}/E_{T}}{M\,O\,I} \end{array}\right)$$

When evaluating the equilibria *e* = (*B*^∗^, 0) on *J* we get:

(26) $$J\,\left(B^{*},0\right)=\left(\begin{array}{cc} 1 & \left(B_{T}-B^{*}\right)\,\frac{P_{a}}{F_{T}}\\ 0 & 1-\frac{F_{T}/E_{T}}{M\,O\,I} \end{array}\right)$$

where it can be seen that *J*(*e*) is a triangular matrix, so its eigenvalues are on the diagonal as: λ_1_ = 1 y $\lambda_{2}=1-\frac{F_{T}/E_{T}}{M\,O\,I}$. Both of the eigenvalues have a positive real part, establishing that the obtained equilibria *e* will have unstable nodes, that is, the long-term dynamics of the system will tend to favor the total infection of bacteria.

#### Simulations and Real Data

We used the software Matlab (The MathWorks, R2016b) to simulate different scenarios for phage adsorption and compared them with the real data, considering the previously measured parameters for the phage PvB and VO.

#### Simulation Varying Parameters

**FIGURE 5:**
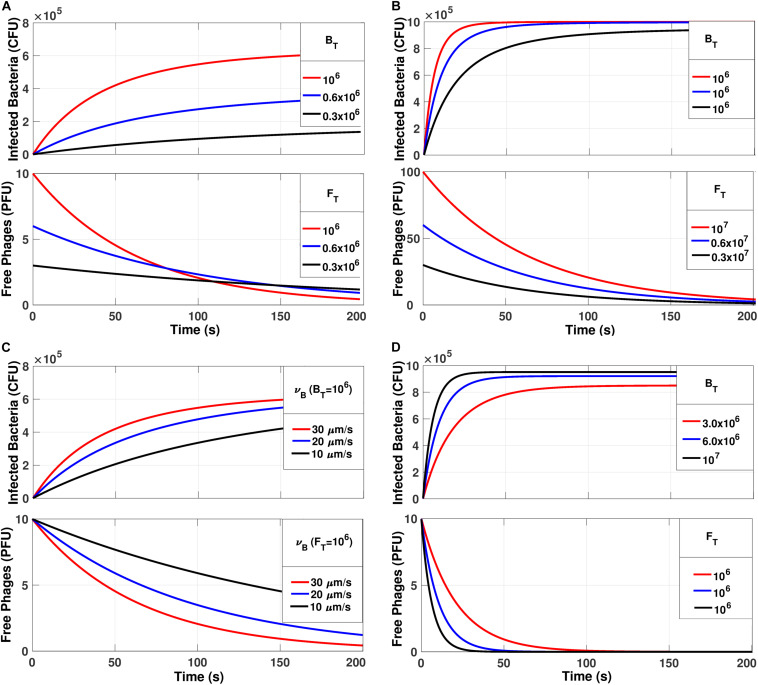
The simulations generated from the Eq. System for *Bt*_*k*_ and *F*_*t_k_*_. **(A)** Increasing the number of bacteria and phages without varying the MOI = 1. **(B)** Keeping the bacterial number constant at 10^6^ CFU and varying the MOI. **(C)** Keeping B_*T*_ = 10^6^ CFU and MOI = 1 and varying υ_*B*_. **(D)** Varying B_*T*_ and keeping F_*T*_ = 10^6^ PFU/mL.

Default parameters were *V*_*B*_ = 5.24 × 10^–10^ mL; *V*_*T*_ = 1 mL; Δ*l*_*B*_ = 1 μm; υ_*B*_ = 30 μm/s; *B*_*T*_ = 10^6^ CFU; *F*_*T*_ = 10^6^ PFU. Using Eq. System 3, the simulations were performed considering default parameters and modifying *B*_*T*_, *F*_*T*_, *MOI*, and υ_*B*_ as shown in Figure 5.

The simulations in Figure 5 were performed with Eq. system 3 and they showed the number of infected bacteria and the number of free phages with respect to time. Besides, the free phages reach zero in all simulations. In Figure 5A, the adsorption was shown to keep the MOI = 1 but changes both the total number of phages and bacteria present in the medium. It was observed that the adsorption curves depend on the amount/number of phages and bacteria present in the community. Therefore, as the total number of individuals present (phages and bacteria) increased, the adsorption slope increased. This establishes that as the dynamics are controlled by the time required for all phages to be adsorbed, then not all bacteria will become infected when MOI = 1. We performed a second simulation varying the MOI, but kept the number of bacteria constant at 106 CFU (Figure 5B).

The model predicted that an infection of the entire bacterial population would occur only with an MOI of ten or more. This is in agreement with our stability result that indicates that the total infection is reached in the equilibrium *e* = (*B*^*T*^, 0). On the other hand, increasing this value does not imply a significant increase in adsorption time and dynamics. The effect of the variation of the bacterial velocity was also simulated (Figure 5C), in which case, the adsorption time was reduced with higher bacterial velocity. Finally, the effect of reducing the number of bacteria present in the medium was simulated, keeping the number of phages constant at 10^6^ PFU/mL (Figure 5D). In this case, it was observed that by decreasing the concentration of bacteria in the medium, it was not possible to reach the equilibrium *e* = (*B*^*T*^, 0). In summary, the MOI could explain the total number of infected bacteria, that is, only if MOI = > 10, all bacteria were infected, and the equilibrium *e* = (*B*^*T*^, 0) were achieved, which would be the theoretical prediction of stability.

#### Model Comparison With Real Data

To evaluate the predictability offered by the model, we compared *in-vitro* data from the initial adsorption of PvB phage on VO with simulations of the same, including, a cubic polynomial regression model of the data as an external modeling source. We considered the default parameters with two different initial phage concentrations, 800 and 8000 PFU.

**FIGURE 6:**
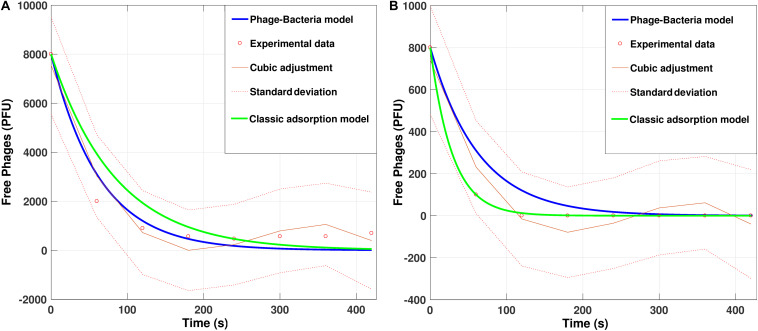
Simulation for adsorption dynamics of the phage PvB on *Vibrio ordalii* versus experimental data using default parameters with **(A)** 8000 phages and **(B)** 800 phages. Cubic adjustment (with R = 0.9560 for **(A)** and R = 0.9356 for **(B)** and classical model curves (using the methodology proposed by Hyman and Abedon, 2009) were included for comparison.

The cubic adjustment made with the real data gave us a regression model $Y=\sum_{i=0}^{3}a_{i}\,x^{i}$ (with *a*_*i*_ determined parameters) that explains 90% of the variability of the data, measured with the coefficient of determination *R*^2^. The construction of the fit model provided us with a reliable tool for comparison with our proposed phage-bacteria absorption model (Figure 6).

## Discussion

Bacteriophages are the most abundant organisms in the biosphere (Clokie et al., 2011; Aziz et al., 2015). Phages cohabit with their host bacteria in an equilibrium state, modulated by both phage infection rate and bacterial resistance mechanisms (Bohannan and Lenski, 2000; Wei et al., 2010; Bull et al., 2014). This is comparable to predator-prey dynamics, but in this case the encounters are governed by probability. In the present work, we built a mathematical model using the major factors determining the initial encounter between phages and bacteria in the function of time. This was done to predict the number of infected bacteria and remnant free phages. At first, we included the amounts of bacteria and phages as determining factors in concordance with previous reports (Yassky, 1962; Murray and Jackson, 1992; Moldovan et al., 2007). However, taking into account the environmental dynamics, we included the living space as a function of cell size and the bacterial velocity, in a probabilistic function that allowed for predicting such encounters more accurately. The data required to feed the model was easily obtainable from simple laboratory experiments (Echeverría-Vega et al., 2019) or public databases, (i.e., BioNumbers database) (Milo et al., 2010). The model assumes that the phage movement occurs by simple diffusion (Yassky, 1962; Gani, 1965; Schwartz, 1976) at approximately 5 × 10^8^ cm^2^s^–1^ (Murray and Jackson, 1992), which is negligible compared to the VO velocity of 30 μm s-1. Additionally, it considers the bacterial size and its vital space or slot, which we defined as all the volume that the bacterium is capable of reaching when it is in a determined space in a specific period of time. Nevertheless, we did not consider the phage size, since it is three orders of magnitude lower, so it does not affect the vital space of the bacteria in normal conditions. In this way, it is possible to generate infection curves for phages and bacteria and therefore predict the number of adsorbed phages and infected bacteria over time. Some existing models consider phage infection as a simplified process based on the mass-action kinetics theory by means of an empirically determined factor called adsorption constant k (Krueger, 1931; Stent, 1963) without taking into account temporal dynamics. A novel model proposed by Joiner et al. (2019) considers bacterial movement and medium effect in the collision frequency. However, our design differs from the previously mentioned research as our model is capable of predicting the sustained decrease of free phages and the number of infected bacteria over time considering temporary changes in the probabilistic functions that modulate the curves. The results obtained from our model show that the principal factors in the initial adsorption in the phage-bacteria binary interaction, are the number and speed of host bacteria. Other environmental factors, like community complexity and habitat components were not included in our model.

Figure 5B shows that an MOI < 10 is not sufficient for the infection of all the bacteria present due to the occurrence of multiple infections, which eliminates the phages from the system before the time required to infect all bacteria has been reached. Nonetheless, one can see in Figure 5A that the concentration of bacteria and phages, while maintaining a constant MOI, causes a considerable effect on the curves, which means the MOI must not be taken as the only factor that modulates phage infections. On the other hand, the increase in speed and/or bacterial concentration significantly decreases the infection time (Figures 5C,D) which means that when the bacteria are highly active they are more likely to be infected by phages in less time (a kill the winner strategy), favoring the establishment of equilibrium states in complex environmental systems (Thingstad, 2000). The experimental validations performed based on the temporal dynamic model were shown to be highly concordant with those predicted. The importance of the present study lies in the generation of a model capable of describing the dynamics of the initial phage infection stage, which is crucial information for designing treatment strategies through phage therapy. Our work is based on statistical predictions using measurable physical parameters of phages and bacteria and hence does not require prior experimental adsorption tests like the classical model (Hyman and Abedon, 2009). Our model has the potential to be used as a basis for modeling both phage-bacteria interactions as well as other natural processes that involve random particle collisions.

## Conclusion

Our model uses statistics to predict the dynamics of the phage-bacterium adsorption process in aquatic environments. It does not require data from previous adhesion experiments and can make predictions using simple measurements of speed, volume, and number of particles. The model is useful in predicting both the number of bacteriophages adsorbed on their bacterial host and the number of infected bacteria in a given specific time. The comparison with experimentally obtained curves demonstrates that the model is applicable in explaining these dynamics. The model was conceived to predict the effectiveness of inoculation in phage therapy, but is also suitable for other physical processes based on particle collision.

## Data Availability Statement

The raw data supporting the conclusions of this article will be made available by the authors, without undue reservation.

## Author Contributions

AE-V and RA conceived the case study. PM-V, CS-S, and AE-V performed the experimental research. AE-V, MA, RD-V, and MS performed and tested the mathematical model. AE-V, FG, and RA checked and discussed the experiment results and wrote the manuscript. All the authors read and approved the final manuscript and critically reviewed the document. All authors contributed to the article and approved the submitted version.

## Conflict of Interest

The authors declare that the research was conducted in the absence of any commercial or financial relationships that could be construed as a potential conflict of interest.
